# The interactions of Lipoprotein(a) with common cardiovascular risk factors in cardiovascular disease risk: evidence based on the UK Biobank

**DOI:** 10.1016/j.ajpc.2025.101008

**Published:** 2025-05-22

**Authors:** Linjun Ao, Raymond Noordam, J Wouter Jukema, Diana van Heemst, Ko Willems van Dijk

**Affiliations:** a Department of Human Genetics, Leiden University Medical Center, Leiden, the Netherlands; b Department of Clinical Epidemiology, Leiden University Medical Center, Leiden, the Netherlands; c Health Campus The Hague/Department of Public Health and Primary Care, Leiden University Medical Center, The Hague, The Netherlands; d Department of Cardiology, Leiden University Medical Center, Leiden, the Netherlands; e Netherlands Heart Institute, Utrecht, the Netherlands; f Department of Internal Medicine, Section of Gerontology and Geriatrics, Leiden University Medical Center, Leiden, the Netherlands; g Department of Internal Medicine, Division of Endocrinology, Leiden University Medical Center, Leiden, the Netherlands; h Einthoven Laboratory for Experimental Vascular Medicine, Leiden University Medical Center, Leiden, the Netherlands

**Keywords:** Lipoprotein(a) risk, Interaction effects, Common cardiovascular risk factors, Coronary artery disease, Calcific aortic valve stenosis

## Abstract

**Background:**

**:** Although Lipoprotein(a) (Lp(a)) is associated with cardiovascular disease, it is unclear whether the associated risk is similar in the presence of other concomitant risk factors. Here, we aimed to investigate the interactions between Lp(a) and common cardiovascular risk factors on coronary artery disease (CAD), calcific aortic valve stenosis (CAVS) and ischemic stroke (IS).

**Methods:**

**:** We included 127,958 unrelated European-ancestry participants from UK Biobank (54.7 % women) with data available on Lp(a) and without a baseline history of CAD, CAVS and IS. Multivariable-adjusted Cox proportional hazards interaction models were used to study whether the associations of Lp(a) with outcomes varied based on the level of total cholesterol (Total-C), low-density lipoprotein-cholesterol (LDL-C), triglycerides (TG) and other cardiovascular risk factors.

**Results:**

**:** Higher Lp(a) levels were associated with higher risks of CAD, CAVS and IS. Per 10 mg/dL increase in Lp(a), hazard ratios [95 % confidence interval] were 1.05 [1.04, 1.06], 1.06 [1.04, 1.09], and 1.01 [0.99, 1.03] for CAD, CAVS and IS, respectively. For CAD, interactions were observed between Lp(a) and Total-C (*P_interaction_*=0.001), LDL-C (*P_interaction_*=4e-4) and TG (*P_interaction_*=0.026). In more detail, participants with Lp(a) ≥ 50 mg/dL in the highest quartile of Total-C, LDL-C and TG showed evidence of additive interaction in CAD, with relative excess risk due to interaction (RERI) of 0.42 (0.17, 0.67), 0.44 (0.18, 0.71), and 0.39 (0.12, 0.67), respectively. No such interactions were observed in CAVS and IS.

**Conclusions:**

Lp(a)-associated CAD risk seems to particularly affect those having levels of Total-C, LDL-C and TG above the thresholds from clinical guidelines.

## Graphical abstract

Central Illustration: The associations of lipoprotein (a) with cardiovascular diseases, and the joint effects between lipoprotein (a) and other lipids on the risk of coronary artery disease. Abbreviations: Lp(a), lipoprotein (a); Total-C, total cholesterol; LDL-C, low-density lipoprotein-cholesterol; TG, triglyceride.

**Figure:**
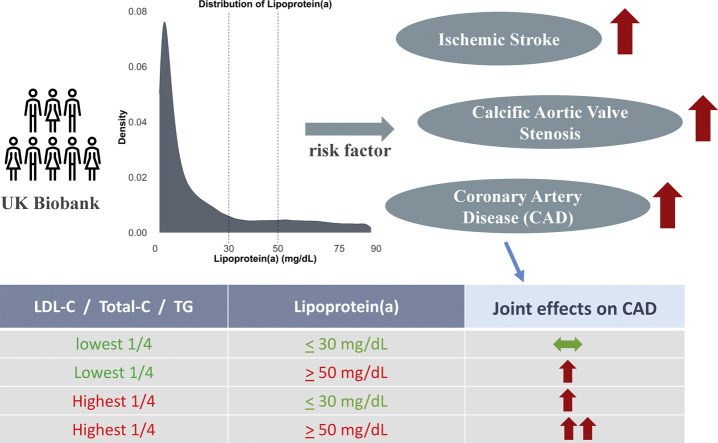

## 1 Introduction

Lipoprotein(a) [Lp(a)], first identified in 1963 [1], is a low-density lipoprotein (LDL)-like particle characterized by a covalently bound apolipoprotein(a) molecule and its blood levels are predominantly determined by genetic factors [2]. The proportion of individuals with Lp(a) ≥50 mg/dL ranges from 31 % for Mexican individuals to 63 % for non-Hispanic-Black individuals [3]. According to current guidelines, Lp(a) levels higher than 50 mg/dL are regarded as a cardiovascular risk-increasing factor [[4], [5], [6], [7]]. Both prospective and genetic evidence supports the associations of elevated Lp(a) with coronary artery disease (CAD) [[7], [8], [9]], and ischemic stroke (IS) [10,11]. In addition, Lp(a) has recently been identified as a risk factor for calcific aortic valve stenosis (CAVS) [[12], [13], [14], [15], [16]].

Except for lipoprotein apheresis, which is the only approved intervention for individuals with elevated Lp(a) [17], there are currently no approved drug therapies for lowering Lp(a). It is thus essential to know whether Lp(a) has homo- or heterogenous effects in subgroups with different risk levels defined by the concomitant presence of other common cardiovascular risk factors. This knowledge could benefit individuals with high Lp(a) to minimize Lp(a)-associated risk through effective control of these modifiable risk factors. In addition, novel Lp(a)-lowering agents are in development, including antisense oligonucleotides and small interfering RNAs, which have shown effectiveness in Lp(a) reduction in clinical trials [18,19]. However, it remains unclear whether Lp(a)-lowering therapies provide similar benefits in all patients, whether the effect is also observed on clinical outcomes, or whether their effectiveness in disease risk reduction depends on baseline lipid profiles, metabolic status, or other risk factors. Examining interactions between Lp(a) and additional cardiovascular risk factors may provide evidence for interventions tailored to individuals who are likely to benefit most.

Previous studies have mainly focused on the interaction effects of Lp(a) and low-density lipoprotein-cholesterol (LDL-C) on atherosclerotic cardiovascular diseases, but showed inconsistent findings [[20], [21], [22], [23], [24]]. However, in addition to LDL-C, other risk factors, such as body mass index (BMI) and blood pressure, are also important risk factors for cardiovascular disease [25,26]. Nevertheless, the interactions between Lp(a) and these risk factors have not been studied in detail. Therefore, the present study aimed to investigate the association of Lp(a) with CAD, CAVS and IS, and the interaction effects of Lp(a) with other common cardiovascular risk factors, including sex, age, family history, smoking, BMI, lipids, glycated haemoglobin (HbA1c) and blood pressure.

## 2 Methods

### 2.1 Study population and design

The present study was embedded in the prospective UK Biobank (UKB) cohort, which recruited 502,628 participants aged 40-70 years across the entire United Kingdom during the baseline survey between 2006 and 2010. Extensive phenotypic and genotypic details of the participants have been collected since the baseline assessment, including sociodemographic data, lifestyle, physical measures, biological samples (blood, urine and saliva), genome-wide genotyping, and prospective follow-up on a wide range of health-related outcomes, etc. The UKB cohort study was approved by the North-West Multicentre Research Ethics Committee (MREC). All participants provided electronic written informed consent for the study. A detailed description of the UKB cohort study has been presented elsewhere [27].

To minimize population stratification bias, the present study restricted participants to 315,585 unrelated individuals with European ancestry, based on the estimated kinship coefficients for all pairs and the self-reported ancestral background [28]. Besides, 75,916 individuals with missing values of Lp(a) were excluded. Another 32,630 participants were excluded due to being diagnosed with any the three examined diseases and/or the use of cholesterol-lowering medication at baseline. Participants (n = 550) with a history of rheumatic fever or rheumatic heart disease were also excluded. After excluding participants with missing data on covariates, we ultimately included 127,958 participants. A flowchart displaying the inclusion process of study participants is provided in Fig. S1.

### 2.2 Lipoprotein (a) measurement

The concentrations of serum Lp(a) were measured in nanomoles per liter (nmol/L) by immunoturbidimetric analysis on the Beckman Coulter AU5800 Platform (Randox Bioscience, UK). To facilitate comparison with prior studies, the unit of Lp(a) was converted from nmol/L divided by 2.15 to milligrams per deciliter (mg/dL) [29].

### 2.3 Other investigated variables

The present study investigated 12 common cardiovascular risk factors, which were collected and measured at the moment of study enrolment. Information on sex, age, family history of heart disease and smoking status was collected based on questionnaire. BMI was calculated from height and weight measured during the baseline assessment at one of the study centres. Lipids, including total cholesterol (Total-C, mmol/L), LDL-C (mmol/L), high-density lipoprotein cholesterol (HDL-C, mmol/L), and triglycerides (TG, mmol/L), were measured based on blood samples with the Beckman Coulter AU5800 platform. HbA1c (mmol/mol) was measured by HPLC analysis on a Bio-Rad VARIANT II Turbo based on blood samples collected during baseline. In addition, blood pressure was measured twice in a resting sitting position at the study centre, and the average of the two measurements was used. Correcting blood pressure in participants taking antihypertensive medication was found to improve analyses and hence the power of epidemiological studies compared to no medication adjustment or the exclusion of treated individuals [[30], [31], [32], [33]]. In agreement with previous studies, including genomics consortia that aimed to identify genetic variants associated with blood pressure measures [34], if participants reported taking antihypertensive medication, 10 and 5 mmHg were added to the average measured systolic and diastolic blood pressure, respectively.

To assess and visualize effect modifications by the above 12 risk factors in subsequent stratified analyses, participants were categorised according to each risk factor: sex groups (female, male), three age groups [40,50), [50, 60), [60, 70] years), with or without a family history of heart disease (yes/no), three groups of smoking status (never, previous, current), quartile groups for Total-C, LDL-C, HDL-C, and TG, three groups for BMI (<25, [25, 30), ≥ 30 kg/m^2^), HbA1c (<42, [42, 48), ≥ 48 mmol/mol), systolic blood pressure (<120, [120, 140), ≥ 140 mmHg) and diastolic blood pressure (<80, [80, 90), ≥ 90 mmHg).

Other covariates to be adjusted for in subsequent analyses, including Townsend Deprivation index, alcohol consumption frequency, and physical activity, were measured and collected during the baseline survey.

### 2.4 Cardiovascular disease outcomes

According to the International Classification of Diseases edition 10 (ICD-10), CAD is defined as angina pectoris (I20), myocardial infarction (MI) (I21 and I22), and acute and chronic ischemic heart disease (IHD) (I24 and I25); CAVS was defined as one of the nonrheumatic aortic valve disorders (I35.0); IS is defined as cerebral infarction (I63). These variables have been generated by the UKB data management team through a standard algorithm (https://biobank.ndph.ox.ac.uk/showcase/ukb/docs/first_occurrences_outcomes.pdf), combining self-reported health conditions from baseline and linked data from hospital admissions, primary care, and death registers. The linked data and its sources were presented here (https://biobank.ndph.ox.ac.uk/showcase/exinfo.cgi?src=Data_providers_and_dates). Outcomes in the analysis were incident disease during the time period from recruitment to October 31st, 2022. Follow-up time is computed from the baseline visit to the occurrence of the disease event, death, loss-to-follow-up or the end of follow-up, whichever came first.

### 2.5 Statistical analysis

#### 2.5.1 Main analyses

Baseline characteristics of the included study population were presented as mean (standard deviation, SD) or median (interquartile range, IQR) for continuous variables and frequency (proportion) for categoric variables. The associations of Lp(a) levels with incident CAD events, CAVS events, and IS events were examined using Cox proportional hazards models adjusted for sex, age, Townsend Deprivation index, smoking status, alcohol consumption frequency, and physical activity.

The interaction effects, on a multiplicative scale, were assessed by testing the interaction term between Lp(a) and each of the 12 risk factors using multivariable-adjusted Cox proportional hazards models with covariates of sex, age, Townsend index, smoking status, alcohol consumption frequency, and physical activity. After grouping individuals based on the categorical variables of each risk factor, stratified analyses were conducted to investigate the effect modifications by those risk factors. The heterogeneity among different strata was assessed using the χ^2^ test. A *P* value for the interaction term below 0.0042 (0.05/12, where 12 represents the number of examined risk factors) was considered as statistically significant with Bonferroni correction for multiple testing.

Furthermore, previous international guidelines identified Lp(a) levels ≥ 50 mg/dL as a cardiovascular risk-increasing factor and suggested Lp(a) < 30 mg/dL as a threshold to rule out risk, with 30-50 mg/dL representing an intermediate ‘grey zone’ [[4], [5], [6]]. Therefore, for the identified significant multiplicative interactions on the corresponding diseases, we grouped participants according to the combination of the suggested Lp(a) cut-offs (i.e., < 30 mg/dL, 30-50 mg/dL, ≥ 50 mg/dL) and categorical risk factors. The joint effects of Lp(a) and risk factors on the corresponding disease were then estimated using participants with Lp(a) <30 mg/dL and the lowest level of risk factor as the reference group. We further tested the additive interaction by calculating the relative excess risk due to interaction (RERI) and the attributable proportion (AP) [35,36].

#### 2.5.2 Sensitivity analysis

In line with the principles of triangulation of findings done in observational studies [37], we explored the associations of Lp(a) with CAD, CAVS, and IS in two-sample Mendelian randomization (MR) analyses. A total of 43 genetic variants with a linkage < 0.4 in the *LPA* gene region were found to be conditionally and significantly (*P* < 5e-8) associated with Lp(a) levels in data sets external to the UKB (Table S1) (38). Summary association statistics of the 43 genetic variants with each outcome were estimated or extracted from large databases, namely CARDIoGRAMplusC4D [39] and MEGASTROKE [40] for CAD and IS, respectively, and UKB and FinnGen study [41] for all three outcomes. Detailed MR methods, including the UKB genotyping data, can be found in the supplementary material. Estimates by two-sample MR were expressed as odds ratios (ORs) for per 10mg/dL increase in Lp(a) levels.

We also investigated the interaction between the *LPA* genetic risk score (*LPA* GRS) and the 12 risk factors as the sensitivity analyses using multivariable-adjusted Cox proportional hazards models with the covariates of sex, age, and the first ten genetic principal components (PCs). The *LPA* GRS was scaled and calculated by adding the number of effect alleles of the above mentioned 43 genetic variants, weighted by their corresponding effects on Lp(a) [38]. The *LPA* GRS explained about 57.7 % of the variance in Lp(a) levels among the included participants in the present study. The risk of *LPA* GRS in subgroups defined by risk factors were assessed using stratified analysis. However, collider bias would be introduced when conducting conditional analysis on a variable that is directly affected by two other variables or is a causal descent of a collider in a causal diagram [42]. In line with this principle, and the evidence that Lp(a) associates with metabolic factors (e.g., lipids) [43,44], these metabolic risk factors in the present study could be colliders (Fig. S2). Therefore, based on the method proposed by Coscia et al. [42], we constructed the residual colliders, which were derived from the regression of the potential colliders (BMI, Total-C, LDL-C, TG, HDL-C, HbA1c, SBP and DBP) on the *LPA* GRS, and then estimated the risk of *LPA* GRS in strata defined by quantiles of the residual colliders. In addition, consistent with previous studies [45,46], we additionally corrected the measured Total-C and LDL-C concentration by subtracting the contribution of the cholesterol content from Lp(a), denoted as Total-C_cor and LDL-C_cor. All of the above analyses conducted for Total-C and LDL-C were repeated for Total-C_cor and LDL-C_cor.

Results based on the multivariable-adjusted Cox proportional hazards models were expressed as hazard ratios (HR) and 95 % confidence intervals (95 % CI). All statistical analyses were performed using R software (version 4.3.1).

## 3 Results

### 3.1 Baseline characteristics of study population

The study characteristics are presented in Table 1 stratified by sex. The final study population encompassed 127,958 unrelated European-ancestry participants, with 54.7 % women and a median age of 56 [IQR: 49, 62] years. The median levels of Lp(a) were 9.75 [IQR: 4.51, 27.78] mg/dL and 8.56 [IQR: 4.14, 27.30] mg/dL in women and men, respectively.

**Table 1:** Baseline characteristics of the included population stratified by sex.

	Overall	Women	Men
n	127958	69944	58014
Age (median [IQR]	56.00 [49.00, 62.00]	56.00 [49.00, 62.00]	56.00 [49.00, 62.00]
Sex = Male ( %)	58014 (45.3)	0 (0.0)	58014 (100.0)
Townsend index (mean (SD))	-1.60 (2.91)	-1.60 (2.87)	-1.60 (2.95)
Family history of HD = Yes ( %)	47980 (37.5)	28057 (40.1)	19923 (34.3)
Smoking status ( %)			
Never	72171 (56.4)	41579 (59.4)	30592 (52.7)
Previous	43099 (33.7)	22538 (32.2)	20561 (35.4)
Current	12688 (9.9)	5827 (8.3)	6861 (11.8)
Lpa, mg/dL (median [IQR]	9.20 [4.33, 27.56]	9.75 [4.51, 27.78]	8.56 [4.14, 27.30]
BMI, kg/m^2^ (mean (SD))	26.77 (4.45)	26.36 (4.79)	27.26 (3.95)
Total-C, mmol/L (mean (SD))	5.89 (1.04)	5.98 (1.07)	5.77 (1.00)
Total-C_cor*, mmol/L (mean (SD))	5.80 (1.04)	5.90 (1.06)	5.69 (1.00)
LDL-C, mmol/L (mean (SD))	3.71 (0.80)	3.70 (0.83)	3.71 (0.77)
LDL-C_cor*, mmol/L (mean (SD))	3.62 (0.80)	3.61 (0.82)	3.62 (0.77)
TG, mmol/L (median [IQR]	1.43 [1.01, 2.07]	1.27 [0.93, 1.80]	1.66 [1.16, 2.40]
HDL-C, mmol/L (mean (SD))	1.48 (0.38)	1.63 (0.38)	1.31 (0.31)
HbA1c, mmol/mol (mean (SD))	34.79 (4.29)	34.75 (4.06)	34.83 (4.56)
SBP, mmHg (mean (SD))	136.45 (18.47)	133.45 (18.91)	140.05 (17.24)
DBP, mmHg (mean (SD))	82.03 (10.13)	80.22 (9.91)	84.21 (9.95)

⁎ representing the values of Total-C and LDL-C were corrected by subtracting the contribution of the cholesterol content from Lp(a). Abbreviations: BMI, body mass index; DBP, diastolic blood pressure; HbA1c: glycated haemoglobin; HD: heart disease; HDL-C, high-density lipoprotein cholesterol; IQR, interquartile range; LDL-C, low-density lipoprotein cholesterol; Lp(a), lipoprotein (a); SD: standard deviation; SBP: systolic blood pressure; Total-C, total cholesterol; TG, triglycerides.

With a median follow up of 13.6 [IQR: 12.9, 14.2] years, 7,740 participants developed CAD, 1,132 participants developed CAVS, and 1,469 developed IS, with incidence rates of 469 (95 % CI: 458, 479), 67 (63, 71) and 86 (82, 91) per 100,000 person-years, respectively.

### 3.2 Associations of Lp(a) with cardiovascular diseases

After adjustment for considered confounders, the HRs of per 10 mg/dL increase in Lp(a) were 1.05 (95 % CI: 1.04, 1.06), 1.06 (95 % CI: 1.04, 1.09), and 1.01 (95 % CI: 0.99, 1.03) for the risk of developing CAD, CAVS, and IS, respectively. The associations estimated by the inverse-variance weighted (IVW) method using two-sample MR analysis in each database and their pooled estimates are presented in Fig. S3. Briefly, the pooled estimated ORs (95 % CI) for the effects of per 10 mg/dL increase in Lp(a) on CAD, CAVS and IS risk were 1.06 (95 % CI: 1.05, 1.07), 1.08 (95 % CI: 1.07, 1.10) and 1.02 (95 % CI: 1.01, 1.02), respectively.

### 3.3 Interaction between Lp(a) and other risk factors

**Fig. 1:**
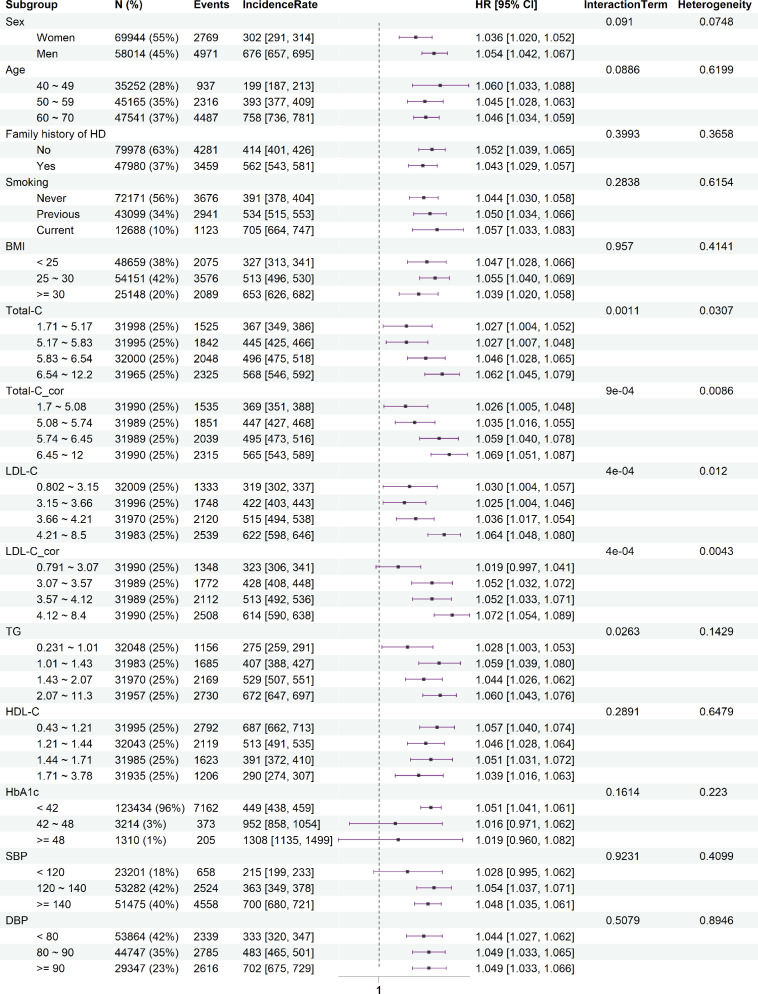
**The interactions between Lp(a) and the common cardiovascular risk factors, and the risk of developing coronary artery disease for per 10 mg/dL increase of Lp(a) in subgroups.** Abbreviations: BMI, body mass index; CI, confidence interval; DBP, diastolic blood pressure; HbA1c: glycated haemoglobin; HD: heart disease; HDL-C, high-density lipoprotein cholesterol; HR, hazard ratio; LDL-C, low-density lipoprotein cholesterol; Lp(a), lipoprotein (a); SBP: systolic blood pressure; Total-C, total cholesterol; TG, triglycerides. LDL-C_cor and Total-C_cor represent the corrected LDL-C and Total-C. The ‘IncidenceRate’ shows the incidence rate of developing CAD per 100,000 person-years in different subgroups. The ‘Heterogeneity’ shows the *P*-values of χ^2^ tests for HRs among subgroups. The ‘InteractionTerm’ shows the *P*-values of tests for the interaction terms between Lp(a) and the original risk factors.

With Bonferroni correction, we observed evidence for interaction of Lp(a) with Total-C (*P_interaction_* [*P* value for the interaction term] = 0.001) and LDL-C (*P_interaction_* = 4e-4) on CAD risk. In addition, we observed an interaction between TG and Lp(a) on CAD risk (*P_interaction_* = 0.026) at a nominal threshold of *P* <0.05 but not after correction for multiple testing. Stratified analyses showed a higher risk of CAD associated with Lp(a) in participants with higher levels of Total-C and LDL-C (Fig. 1). For CAVS risk, the interaction between Lp(a) and sex (*P_interaction_* = 0.0029) was observed, with HRs for per 10 mg/dL increase in Lp(a) being 1.02 (95 % CI: 0.98, 1.06) and 1.09 (95 % CI: 1.06, 1.13) in women and men, respectively (Fig. S4). We did not observe evidence for additional interaction effects for CAVS and IS (Fig. S4 and Fig. S5).

**Fig. 2:**
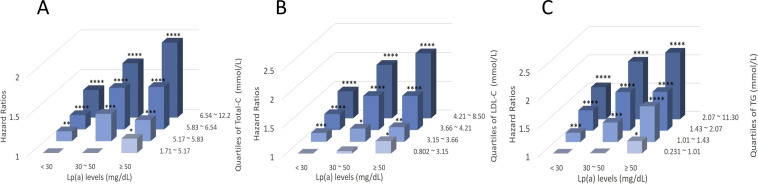
**The joint effects between Lp(a) and (A) Total-C, (B) LDL-C, and (C) TG on coronary artery disease.** LDL-C, low-density lipoprotein cholesterol; Lp(a), lipoprotein (a); Total-C, total cholesterol; TG, triglycerides. Hazard ratios were calculated using Cox proportional hazards regression analysis after adjusting for age, sex, Townsend index, smoking status, alcohol consumption frequency, and physical activity. Individuals with Lp(a) < 30 mg/dL and lowest levels of (A) Total-C, (B) LDL-C and (C) TG are the reference group. * indicated *P* < 0.05, ** indicated *P* < 0.01, *** indicated *P* < 0.001, **** indicated *P* < 0.0001.

The joint effects of Lp(a) with Total-C, LDL-C and TG on CAD risk are presented in Fig. 2, and detailed in Table S2. Compared to the reference group with Lp(a) < 30 mg/dL and lipid concentrations in the lowest quartile, groups with higher Lp(a) and/or higher lipids concentrations showed an increased risk of CAD. The highest CAD risks were found in groups with Lp(a) ≥ 50 mg/dL and Total-C, LDL-C and TG concentrations in the highest quartile, being 1.94, 2.13, and 2.18 times higher, respectively, compared to the reference group. The additive interactions were also observed in the group with Lp(a) ≥ 50 mg/dL and highest quartile levels of Total-C, LDL-C and TG, with RERI (95 % CI) being 0.42 (0.17, 0.67), 0.44 (0.18, 0.71), and 0.39 (0.12, 0.67) respectively, and AP (95 % CI) being 0.21 (0.10, 0.33), 0.21 (0.09, 0.33), and 0.18 (0.06, 0.30) respectively (Table 2).

**Table 2:** The interactions on additive scale between Lp(a) and Total-C, LDL-C, and TG on coronary artery disease in different interaction groups.

		RERI [95 % CI]		AP [95 % CI]	
		30 ≤ Lp(a) < 50 mg/dL	Lp(a) ≥ 50 mg/dL	30 ≤ Lp(a) < 50 mg/dL	Lp(a) ≥ 50 mg/dL
**Total-C (mmol/L)**					
	5.17 ∼ 5.83	0.22 [-0.04, 0.48]	-0.04 [-0.28, 0.2]	0.17 [-0.01, 0.34]	-0.03 [-0.22, 0.16]
	5.83 ∼ 6.54	0.35 [0.08, 0.61]	0.17 [-0.07, 0.41]	0.23 [0.07, 0.38]	0.11 [-0.04, 0.26]
	6.54 ∼ 12.2	0.34 [0.06, 0.63]	0.42 [0.17, 0.67]	0.20 [0.05, 0.35]	0.21 [0.10, 0.33]
**LDL-C (mmol/L)**					
	3.15 ∼ 3.66	0.03 [-0.24, 0.31]	-0.12 [-0.37, 0.14]	0.03 [-0.19, 0.25]	-0.09 [-0.3, 0.12]
	3.66 ∼ 4.21	0.27 [-0.02, 0.56]	0.10 [-0.16, 0.36]	0.17 [0.00, 0.34]	0.06 [-0.1, 0.22]
	4.21 ∼ 8.50	0.42 [0.11, 0.72]	0.44 [0.18, 0.71]	0.22 [0.07, 0.36]	0.21 [0.09, 0.33]
**TG** **(mmol/L)**					
	1.01 ∼ 1.43	0.18 [-0.12, 0.47]	0.25 [-0.01, 0.51]	0.13 [-0.08, 0.34]	0.15 [0.00, 0.30]
	1.43 ∼ 2.07	0.32 [0.02, 0.61]	0.11 [-0.15, 0.37]	0.19 [0.03, 0.35]	0.06 [-0.08, 0.21]
	2.07 ∼ 11.30	0.45 [0.14, 0.76]	0.39 [0.12, 0.67]	0.22 [0.08, 0.36]	0.18 [0.06, 0.30]

Abbreviations: RERI, relative excess risk due to interaction; AP: attributable proportion; CI, confidence interval; Lp(a), lipoprotein (a); LDL-C, low-density lipoprotein cholesterol; Total-C, total cholesterol; TG, triglycerides.

**Fig. 3:**
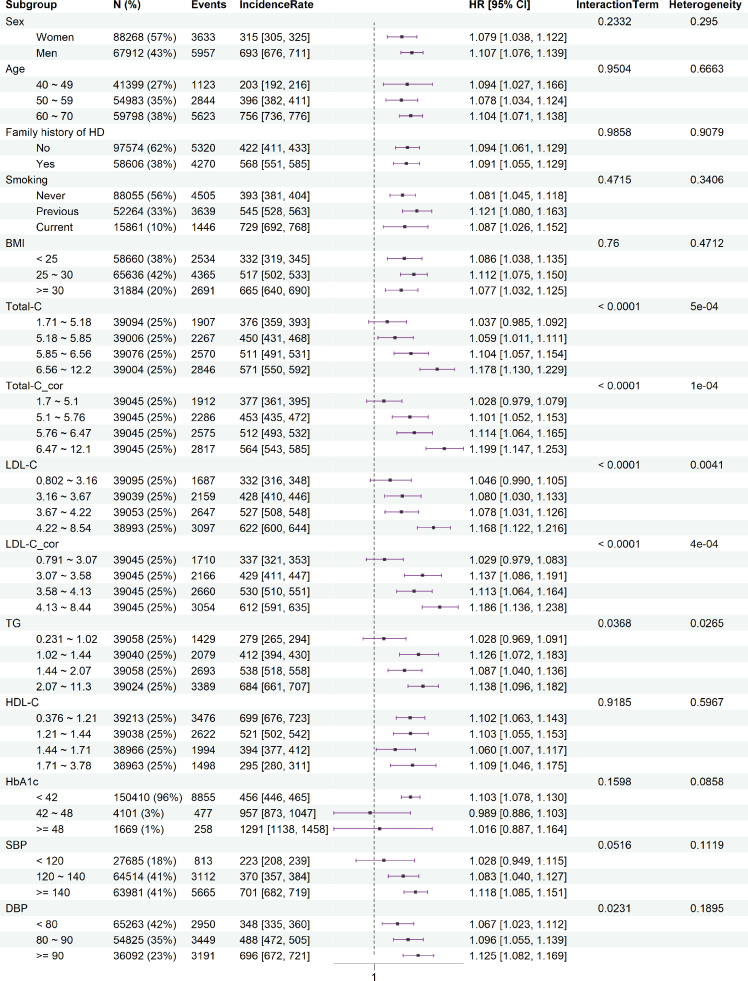
**The interactions between *LPA* GRS and the common cardiovascular risk factors, and the risk of developing coronary artery disease for per one-SD increase of *LPA* GRS in subgroups**. Abbreviations: BMI, body mass index; CI, confidence interval; DBP, diastolic blood pressure; HbA1c: glycated haemoglobin; HD: heart disease; HDL-C, high-density lipoprotein cholesterol; HR, hazard ratio; LDL-C, low-density lipoprotein cholesterol; Lp(a), lipoprotein (a); SBP: systolic blood pressure; Total-C, total cholesterol; TG, triglycerides. LDL-C_cor and Total-C_cor represent the corrected LDL-C and Total-C. The ‘IncidenceRate’ shows the incidence rate of developing CAD per 100,000 person-years in different subgroups. The ‘Heterogeneity’ shows the *P*-values of χ^2^ tests for HRs among subgroups. The ‘InteractionTerm’ shows the *P*-values of tests for the interaction terms between *LPA* GRS and the original risk factors.

The interactions between the *LPA* GRS and examined risk factors on CAD risk showed similar results (Fig. 3) to the findings for measured Lp(a) levels. With increasing levels of Total-C and LDL-C, the risk of developing CAD per one-SD increase in *LPA* GRS also increased. Except for the nominal evidence for the interaction between *LPA* GRS and sex on CAVS (*P_interaction_* = 0.046), no other interactions between *LPA* GRS and these risk factors were detected for CAVS and IS (Fig. S6 and Fig. S7). After considering potential collider bias, the results remained similar to those from the main analyses (Fig. S8-S10). The interaction and stratification results for Total-C_cor and LDL-C_cor were similar to those results for Total-C and LDL-C, respectively (Fig. 1, Fig. 3).

## 4 Discussion

In the present study, using prospective analyses and two-sample MR analyses, we confirmed the associations between elevated Lp(a) levels and the risks of CAD, CAVS and IS, with CAVS in particular showing the largest risk estimates. For CAD, but not for CAVS and IS, Total-C, LDL-C and TG showed evidence for interactions, both on a multiplicative and additive scale, with Lp(a). In particular, participants with higher levels of Total-C, LDL-C and TG had excessively higher Lp(a)-associated CAD risk. Evidence for an interaction between Lp(a) and sex was observed for CAVS risk, with men having a stronger Lp(a)-associated risk of CAVS than women. Evidence for these interactions was further supported using genetically determined Lp(a) levels.

Evidence from population studies support a strong association between Lp(a) and atherosclerotic cardiovascular disease [47]. One previous study presented that using 43 *LPA* genetic variants, each 10 mg/dL lower genetically-predicted Lp(a) was associated with a 5.8 % lower risk of coronary heart disease [38]. Our study not only confirmed the Lp(a)-associated CAD risk in prospective analyses, but also showed a 6 % higher risk of CAD for each 10 mg/dL higher genetically-predicted Lp(a) using the same genetic variants. There is increasing evidence showing Lp(a) as an independent and causal risk factor for CAVS, but most of the previous genetic studies only used two SNPs in the *LPA* locus, i.e., rs10455872 and rs3798220 [[12], [13], [14], [15], [16]], which may have resulted in limited statistical power. Our study, based on all independent SNPs associated with Lp(a) levels in the *LPA* region, robustly showed that elevated Lp(a) associated with higher risk of CAVS, which may even be higher than the risk of Lp(a)-associated CAD risk. However, despite these findings, the clinical benefit of lowering Lp(a) specifically for the examined diseases remains to be proven [16]. Several ongoing clinical trials are expected to provide insight in the clinical benefit of Lp(a) lowering for preventing major adverse cardiovascular events (MACE) and CAVS, such as the HORIZON clinical trial exploring the effect of pelacarsen on cardiovascular events (NCT04023552), the OCEAN(a) outcomes trial for olpasiran (NCT05581303), and the single-ascending dose study for lepodisiran (NCT05565742).

Our study identified a multiplicative interaction between Lp(a) and LDL-C for CAD risk among healthy participants not taking lipid-lowering medication. We found that the lower LDL-C levels, the lower the risk of CAD caused by elevated Lp(a) levels. Most previous observational studies, including those in patients with CAD, support our results, showing that the risk associated with Lp(a) for the first or recurrent cardiovascular incident and mortality was modified by the LDL-C levels [[20], [21], [22], [23]]. Although one study did not find an interaction between Lp(a) and corrected LDL-C levels, it still showed that the risk associated with elevated Lp(a) attenuated when LDL-C levels were less than 2.5 mmol/L in primary prevention [24].

From intervention studies, evidence on the association of Lp(a) with cardiovascular risk for patients taking LDL-C lowering therapy is inconsistent. In the JUPITER trial, for individuals treated with rosuvastatin and with a median LDL-C of 1.4 mmol/L, elevated Lp(a) (≥ 50 mg/dL) was not found to be associated with cardiovascular risk with an HR of 1.67 [95 % CI: 0.93, 3.02] [29]. Another study showed that elevated levels of Lp(a) (≥ 30 mg/dL) were associated with all-cause mortality and acute coronary syndrome, independent of the LDL-C lowering interventions [48]. Proprotein convertase subtilisin/kexin type 9 (PCSK9) inhibitors have been shown to reduce Lp(a) levels by 20-25 % [49], which accounted for 3.8 % of the total beneficial effects of PCSK9 inhibitors on the risk of CAD [50]. Given the effectivity of PCSK9 inhibitors in lowering LDL-C, previous evidence suggests that PCSK9 inhibitors could provide incremental clinical benefit for participants with high levels of both Lp(a) and LDL-C [51]. Notably, consistent with this clinical evidence, our results showed an interaction between Lp(a) and LDL-C on the additive scale. In the group with the highest concentrations of both Lp(a) and LDL-C, 21 % of the CAD risk was attributable to their interaction, whereas no such effect was observed in groups with lower levels of LDL-C. However, the extent to which the effect of Lp(a) on the risk of CAD is modified by the LDL-C levels remains to be firmly established.

Few studies have explored the interaction between Lp(a) levels and other common cardiovascular risk factors [[52], [53], [54]]. In line with these previous studies, we found no evidence of a multiplicative interaction of Lp(a) with BMI, SBP and DBP in the cardiovascular diseases. A previous study further identified an interaction between Lp(a) and BMI for first incident acute myocardial infarction on additive scale with RERI (95 % CI) of 2.45 (0.36, 4.54), but this additive interaction effect was only present at the fifth quintile of Lp(a) (> 246.9 mg/L) [52]. In addition, our study found a multiplicative interaction between Lp(a) and sex in CAVS risk. The important role of sex in the pathophysiology of the calcific aortic valve disease has recently been recognized. Men have a higher incidence of calcific aortic valve disease, with higher levels of calcification for the same degree of hemodynamic stenosis when compared to women [55,56]. Our results showed a stronger association between Lp(a) and CAVS risk in men than in women, aligning with the known sex-specific characteristics of calcific aortic valve disease. This finding suggests that the higher CAVS risk in men may, in part, be driven by Lp(a) [57].

Several structural features of Lp(a) have been proposed to underlie its pathological effects on cardiovascular diseases. Lp(a) not only contains the proatherogenic component LDL-C, but is also a major carrier of oxidized phospholipids, which accumulate in injured vessels and aortic valve leaflets when Lp(a) concentrations are high, leading to local endothelial dysfunction, lipid accumulation, calcification, and inflammation [2]. Specifically, evidence supports a causal association of Lp(a) with atherosclerotic cardiovascular disease and CAVS [2,6,16]. In addition, previous studies have demonstrated causal associations between atherogenic lipids, such as LDL-C and TG, and CAVS [58,59]. However, our study observed the interactions between Lp(a) and lipids only in CAD but not in CAVS, suggesting that the mechanisms linking Lp(a) to CAVS may differ from those linking atherogenic lipids to CAVS.

Furthermore, the association between elevated Lp(a) and IS risk is less well-explored compared to CAD, and large population-based cohort studies have reported conflicting results. However, recent meta-analyses and genetic association studies do support Lp(a) as a risk factor for IS [10,11]. Within our study population, we only observed a weak association between Lp(a) and IS risk based on prospective multivariable-adjusted analyses (HR: 1.01 [0.99, 1.03] and MR analyses (OR: 1.02 [1.01, 1.02]. The inconsistent findings regarding the association between Lp(a) and IS risk may stem from the differences in the location, and underlying pathology of the ischemic stroke event. While elevated Lp(a) levels have been associated with an increased risk of large-artery atherosclerotic stroke [11,60], its role in small-vessel strokes remains less clear. Future studies are required to better understand the associations of different stroke subtypes with Lp(a).

Our study also has several limitations that merit consideration when interpreting the results. First, it remains possible that other unmeasured confounders could be responsible for the higher cardiovascular risks observed when both Lp(a) and other risk factors were elevated. While the present study was conducted in a large study population, our study included a relatively small number of cases, especially for CAVS and IS, resulting in limited statistical power. Additionally, although Lp(a) is primarily genetically determined and remains relatively stable throughout life, certain physiological conditions, such as hormonal changes and thyroid dysfunction, may influence its levels [61]. Our study relied on a single baseline measurement of Lp(a), which does not account for potential temporal variations over time and limits the accuracy of our findings for cardiovascular risk. Furthermore, the present study was, for reasons of sample size, restricted to European-ancestry participants only; interpretation of the present results for other population groups should be done with caution.

In conclusion, the present study confirms the association between Lp(a) and CAD, CAVS and IS. Lp(a)-associated CAD risk particularly affects those having higher levels of Total-C, LDL-C or TG, and the joint effects of high levels of Lp(a) and concurrent hyperlipidemia should not be overlooked. Our findings therefore suggest that it is important to control dyslipidemia for people with high Lp(a) levels.

## Author agreement

All authors have seen and approved the final version of the manuscript being submitted. The manuscript nor parts of it has not been published previously nor are currently under consideration with another journal. There are no conflicts of interest among the authors to disclose.

## CRediT authorship contribution statement

**Linjun Ao:** Writing – review & editing, Writing – original draft, Visualization, Methodology, Formal analysis, Conceptualization. **Raymond Noordam:** Writing – review & editing, Supervision, Methodology, Data curation, Conceptualization. **J Wouter Jukema:** Writing – review & editing. **Diana van Heemst:** Writing – review & editing, Supervision, Conceptualization. **Ko Willems van Dijk:** Writing – review & editing, Supervision, Conceptualization.

## Declaration of competing interest

The authors declare that they have no known competing financial interests or personal relationships that could have appeared to influence the work reported in this paper.

## Appendix Supplementary materials

Image, application 1
