# Supplementary material for: The interactions of Lipoprotein(a) with common cardiovascular risk factors in cardiovascular disease risk: evidence based on the UK Biobank
Source: Am J Prev Cardiol. 2025 May 22;22:101008. doi: 10.1016/j.ajpc.2025.101008 (PMC12162037; doi:10.1016/j.ajpc.2025.101008)
Supplement: Supplementary file 1 [file mmc1.docx]

**Supplementary Materials**

**The interactions of Lipoprotein(a) with common cardiovascular risk factors in cardiovascular disease risk: evidence based on the UK Biobank**

**eMethods**

**Figure S1. Flowchart of eligible participants for analysis in the UK Biobank**

**Figure S2. Directed acyclic graph (DAG) illustrating the (potential) relationship among the examined variables in the present study**

**Figure S3. Causal association between Lp(a) with coronary artery disease, calcific aortic valve stenosis, and ischemic stroke estimated by inverse-variance weighted method for per 10 mg/dL increase in Lp(a)**

**Figure S4.** **The interactions between Lp(a) and the common cardiovascular risk factors, and the risk of developing calcific aortic valve stenosis (CAVS) for per 10 mg/dL increase of Lp(a) in subgroups**

**Figure S5. The interactions between Lp(a) and the common cardiovascular risk factors, and the risk of developing ischemic stroke (IS) for per 10 mg/dL increase of Lp(a) in subgroups**

**Figure S6. The interactions between LPA GRS and the common cardiovascular risk factors, and the risk of developing calcific aortic valve stenosis (CAVS) for per one-SD increase of LPA GRS in subgroups.**

**Figure S7. The interactions between LPA GRS and the common cardiovascular risk factors, and the risk of developing ischemic stroke (IS) for per one-SD increase of LPA GRS in subgroups**

**Figure S8. The risk of developing coronary artery disease (CAD) for per one-SD increase of *LPA* GRS in subgroups defined by residual-risk factors**

**Figure S9. The risk of developing calcific aortic valve stenosis (CAVS) for per one-SD increase of *LPA* GRS in subgroups defined by residual-risk factors**

**Figure S10. The risk of developing ischemic stroke (IS) for per one-SD increase of *LPA* GRS in subgroups defined by residual-risk factors**

**Table S1. Genetic variants used in the MR analysis and *LPA* genetic risk score obtained from Burgess et al. ^(1)^**

**Table S2. The joint effects (hazard ratios [95% CI]) between Lp(a) and LDL-C, Total-C, and TG on coronary artery disease.**

# **eMethods**

## **1. Genotyping and genetic imputations in UK biobank**

UK Biobank has undertaken a project to genotype all 500,000 participants. The majority of participants (~450k) are genotyped on the UK Biobank Axiom^®^ Array, with 50,000 participants genotyped on the Affymetrix UK BiLEVE Axiom^®^ array. The quality control of the genetic data was undertaken at the Wellcome Trust Centre for Human Genetics (WTCHG). Further details of the array design, genotyping and imputation procedures have been described elsewhere (2). In addition, UK Biobank resources performed centralized imputations on the autosomal SNPs using computationally efficient methods combined with the Haplotype Reference Consortium reference panels (3), UK10K haplotype (4), and 1000 Genomes Phase 3 (5) resources. Autosomal SNPs were pre-phased using SHAPEIT3 and imputed using IMPUTE4. In total, ~96 million SNPs were imputed.

## **2. Mendelian randomization**

MR uses genetic variants, typically single-nucleotide polymorphisms (SNPs), as instrumental variables (6, 7). MR studies depend on three main assumptions, notably: 1) the genetic variant must be associated with the exposure; 2) the genetic variant should not be associated with confounders; 3) the genetic variant affects the outcome only through the exposure. This study used the two-sample MR method, which requires that groups of participants in the gene-exposure association analysis and gene-outcome association analysis do not overlap (8-10). The overall workflow of MR analyses is presented below.





### **2.1 Associations of genetic variants with exposure**

The selection of genetic variants for Lp(a) was detailed described by Burgess et al. (1). They started with the full list of 2462 candidate variants in the *LPA* gene region (660kb window), in which 936 variants remained available for analysis after filtering out variants that: were monomorphic across all samples, severely deviated from Hardy-Weinberg equilibrium, or did not have a call rate > 95% in each their included study. Then, based on a strict selection algorithm, a total of 43 genetic variants with a linkage < 0.4 in the *LPA* gene region were found to be conditionally and significantly (P < 5e-8) associated with Lp(a) levels (Table S1).

### **2.2 Associations of genetic variants with outcome**

Summary association statistics of the identified Lp(a)-related SNPs with each outcome were estimated or extracted from different large databases, namely CARDIoGRAMplusC4D (11) and MEGASTROKE (12) for CAD and IS, respectively, and UKB and FinnGen study for all three outcomes.

A total of 313,987 unrelated European-ancestry participants from UKB were included in the MR analyses. Outcomes were prevalent or incident diseases, details of all outcome diagnoses are described in the ‘Cardiovascular Disease Outcomes’ subsection of the main manuscript. In total, 37,597 cases and 276,390 controls for CAD, 4,989 cases and 308,998 controls for CAVS, and 5,846 cases and 308,141 controls for IS were identified. Using the software program of GEM (version 1.4.2) (13), we performed the genome-wide association study (GWAS) to assess the associations of genetic variants with CAD, CAVS and IS, adjusting for age, sex, and the top 10 genetic principle components, and extracted the summary association statistics of the selected 43 Lp(a)-associated genetic variants.

FinnGen is a public-private partnership research project launched in 2017, which covered the whole of Finland and combined genotype data from Finnish biobanks and digital health record data from Finnish health registries (14). In the FinnGen project and based on the ICD-10, major coronary heart disease (CHD), was defined as angina pectoris (I20), myocardial infarction (I21 to I23), ischemic heart diseases (I24 and I25), cardiac arrest (I46), and other cause unknown death or unattended death (R96 and R98); CAVS was defined as aortic (valve) stenosis (I35.0) and aortic (valve) stenosis with insufficiency (I35.2). IS was defined as cerebral infarction (I63) and not specified as haemorrhage or infarction stroke (I64). We extracted summary statistics for CHD and CAVS from the release 9 (<https://r9.risteys.finngen.fi/>), and for IS from the release 7 (<https://r7.risteys.finngen.fi/>). Summary association statistics between the 43 SNPs and the three outcomes were extracted from published FinnGen data freeze 9, which were based on 43,518 cases and 333,759 controls for major CHD, 9,153 cases and 368,124 controls for CAVS, and 16,857 cases and 283,057 controls for IS.

The CARDIoGRAMplusC4D consortium assembled 60,801 cases and 123,504 controls from 48 studies for a GWAS meta-analysis of CAD, which was identified as an inclusive diagnosis of myocardial infarction, acute coronary syndrome, chronic stable angina, or coronary stenosis >50%. 77% of the participants were of European ancestry, 13% of South Asian ancestry, 6% of East Asian ancestry, and smaller samples were Hispanic and African Americans (11). The large-scale MEGASTROKE consortium, launched by the International Stroke Genetics Consortium, releases the summary statistics from the 2018 meta-analysis of Genome-wide Association data in stroke and stroke subtypes with 34,217 IS cases and 406,111 controls (12). We extracted summary association statistics of the 43 SNPs with CAD and IS from CARDIoGRAMplusC4D and MEGASTROKE databases, respectively.

### **2.3 Associations of exposure with outcome**

The associations of Lp(a) with outcome from each database were estimated by the inverse-variance weighted (IVW) method, which combines the Wald ratio estimates (estimated association of genetic variants with outcome divided by estimated association of genetic variants with exposure) for individual genetic variant by a fixed-effect meta-analysis with inverse-variants weights (9, 10). Those estimates were expressed as odds ratios (ORs) for the risk of CAD, CAVS and IS for per 10mg/dL increase in Lp(a). We subsequently conducted meta-analyses for each outcome to pool the estimates from different databases. The heterogeneity of the estimated ORs from different databases for each outcome was represented by *I^2^*, and detected by the Cochran Q test (15).

Given that the IVW method assumes all genetic instruments are valid (e.g., no horizontal pleiotropy), we conducted sensitivity analyses using the weighted-median estimator and the MR-Egger method to assess whether IVW analyses were biased due to horizontal pleiotropy (16-18). Rather than taking a weighted mean of the ratio estimates as in the IVW method, the weighted-median estimator could still provide a consistent estimate of the causal effect even when up to 50% of the identified genetic variants are invalid IVs (16). In contrast to the IVW method, the MR-Egger method does not require a zero horizontal pleiotropy effect, and could detect pleiotropy by the intercept term (under the InSIDE assumption), which when different from zero indicates a bias in the IVW estimation (17, 18).





**Figure S1. Flowchart of eligible participants for analysis in the UK Biobank.**

**
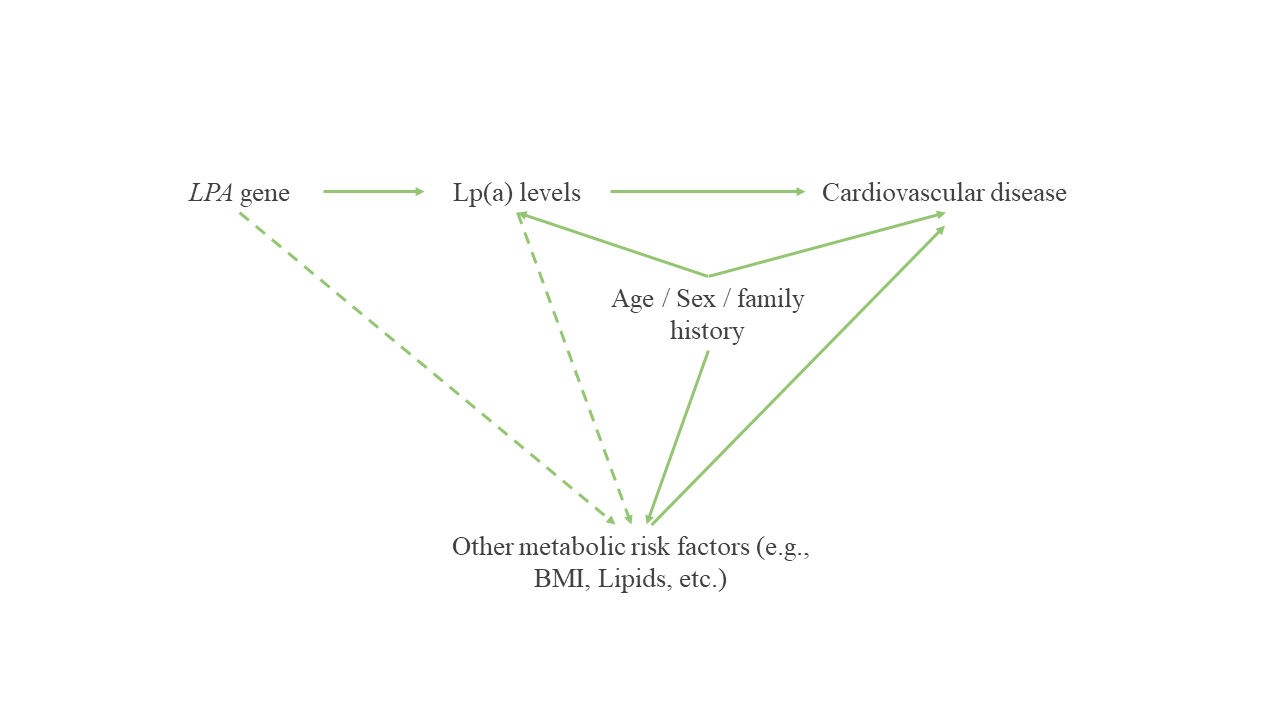
**

**Figure S2. Directed acyclic graph (DAG) illustrating the (potential) relationship among the examined variables in the present study.** The solid lines indicate the existing causal association between two variables. The dashed lines indicate the potential relationship between two variables, which remains to be validated by more future studies. If there was an causal association from Lp(a) to metabolic factors, then collider bias would be introduced by performing stratified analysis on those factors.

**
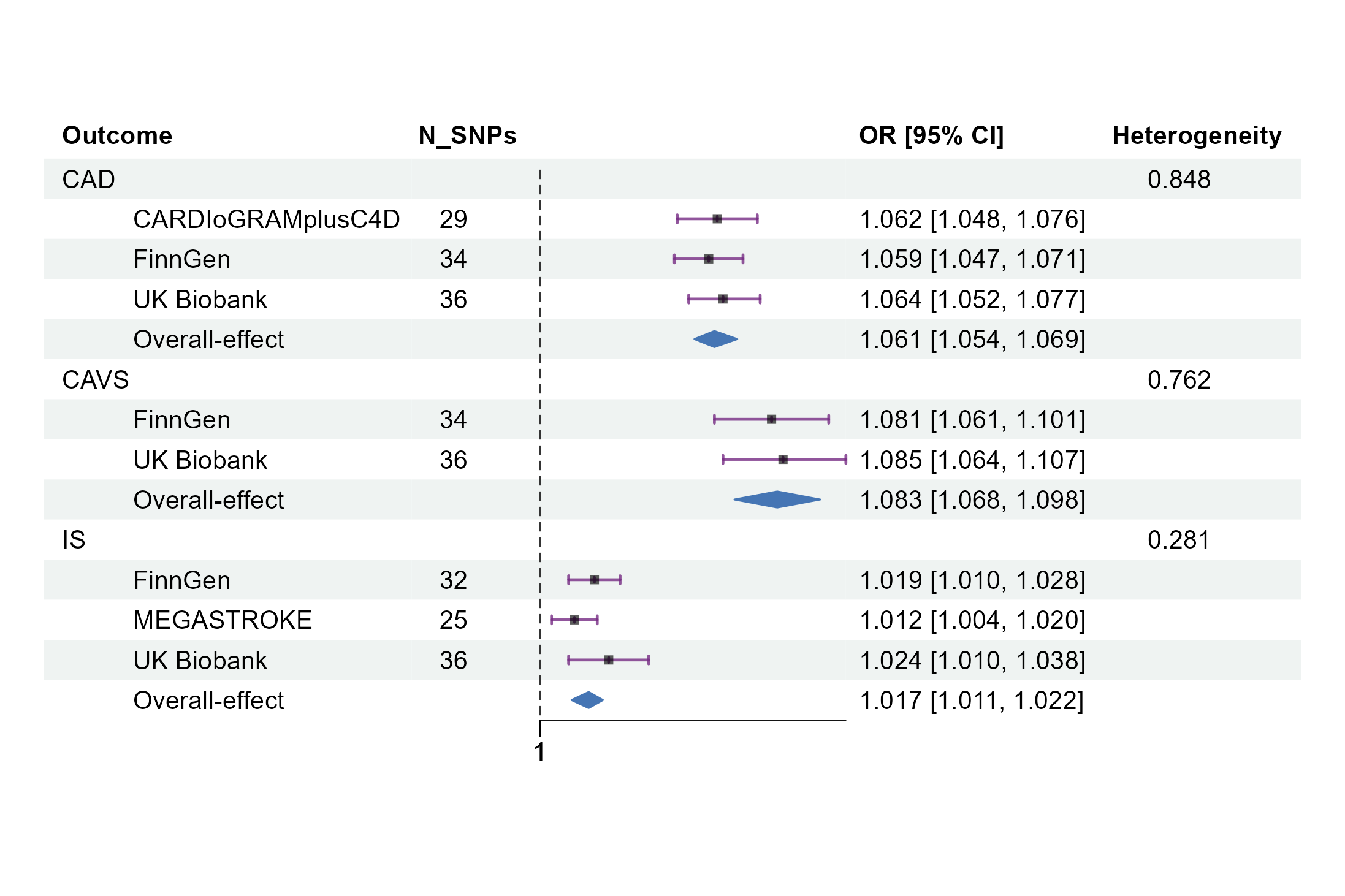
**

**Figure S3. Causal association between Lp(a) with coronary artery disease, calcific aortic valve stenosis, and ischemic stroke** **estimated by inverse-variance weighted method for per 10 mg/dL increase in Lp(a).** CAD: coronary artery disease; CAVS: calcific aortic valve stenosis; IS: ischemic stroke; OR: odds ratio; CI: confidence interval. Estimated ORs were derived from an IVW analysis per outcome database separately, and ‘Overall-effect’ showed the combined ORs across databases using fixed-effect meta-analyses.

**
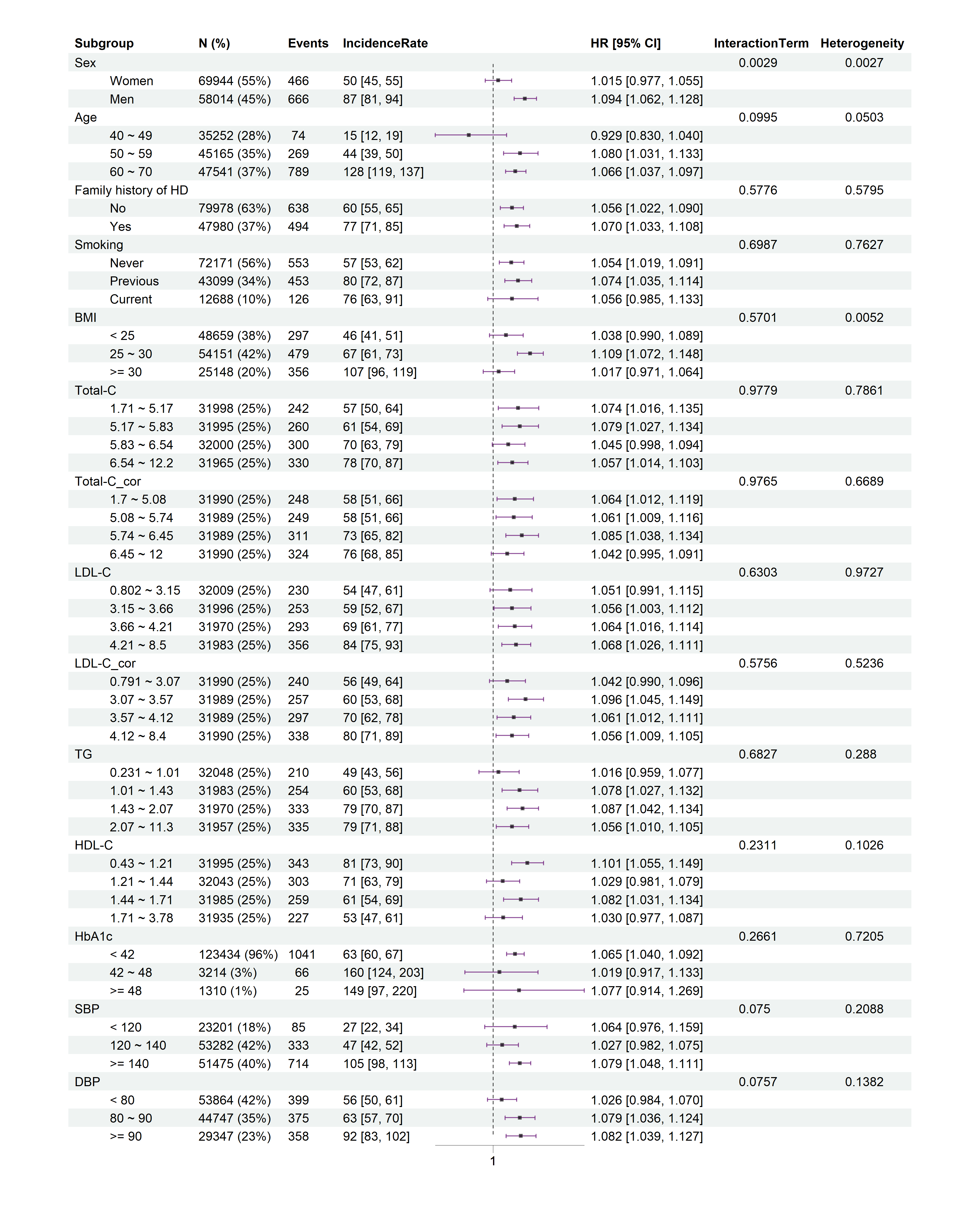
**

**Figure S4. The interactions between Lp(a) and the common cardiovascular risk factors, and the risk of developing calcific aortic valve stenosis (CAVS) for per 10 mg/dL increase of Lp(a) in subgroups.** Abbreviations: BMI, body mass index; CI, confidence interval; DBP, diastolic blood pressure; HbA1c: glycated haemoglobin; HD: heart disease; HDL-C, high-density lipoprotein cholesterol; HR, hazard ratio; LDL-C, low-density lipoprotein cholesterol; Lp(a), lipoprotein (a); SBP: systolic blood pressure; Total-C, total cholesterol; TG, triglycerides. LDL-C_cor and Total-C_cor represent the corrected LDL-C and Total-C. The ‘IncidenceRate’ shows the incidence rate of developing CAVS per 100,000 person-years in different subgroups. The ‘Heterogeneity’ shows the *P*-values of χ^2^ tests for HRs among subgroups. The ‘InteractionTerm’ shows the *P*-values of tests for the interaction terms between Lp(a) and the original risk factors.

**
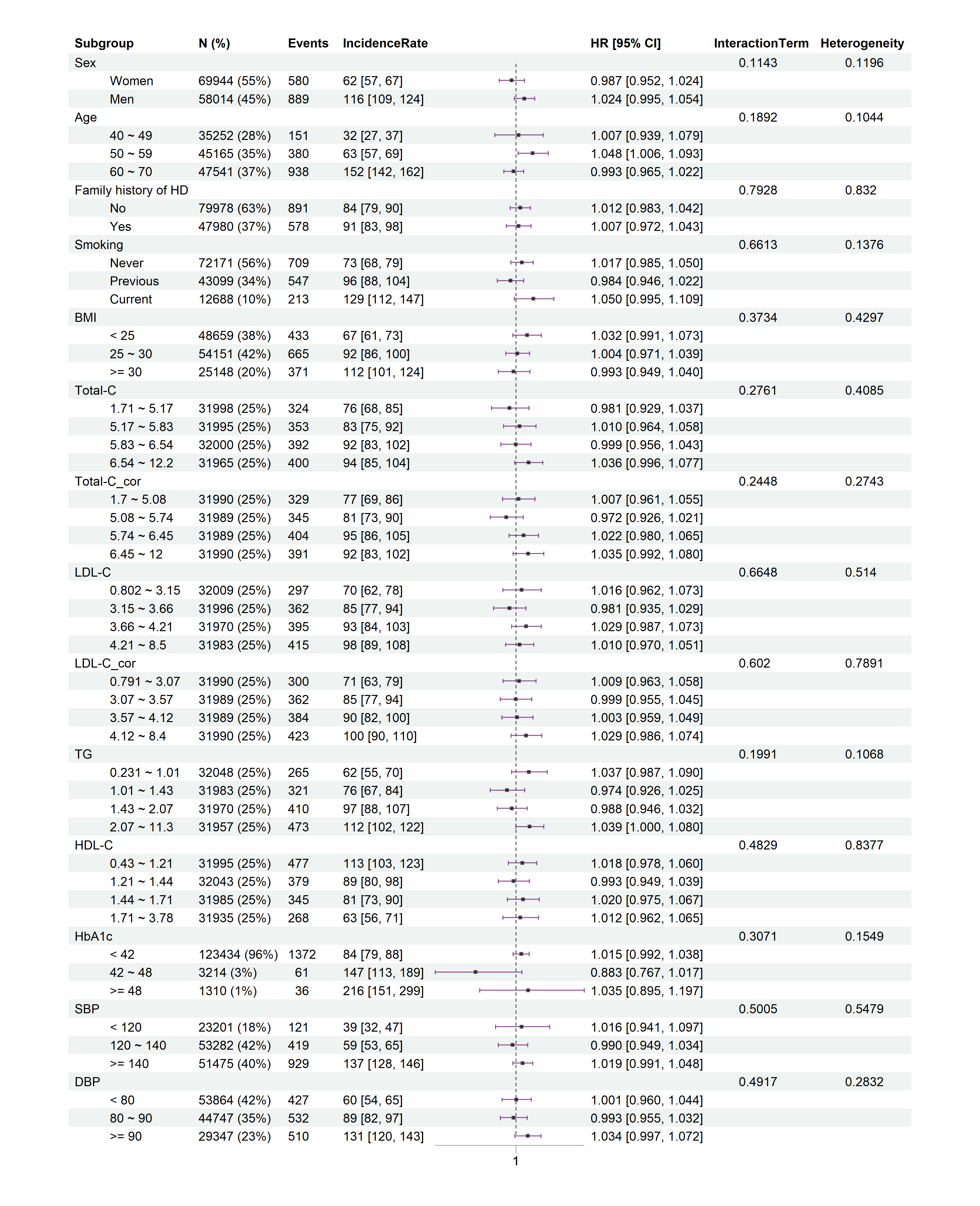
**

**Figure S5. The interactions between Lp(a) and the common cardiovascular risk factors, and the risk of developing ischemic stroke (IS) for per 10 mg/dL increase of Lp(a) in subgroups.** Abbreviations: BMI, body mass index; CI, confidence interval; DBP, diastolic blood pressure; HbA1c: glycated haemoglobin; HD: heart disease; HDL-C, high-density lipoprotein cholesterol; HR, hazard ratio; LDL-C, low-density lipoprotein cholesterol; Lp(a), lipoprotein (a); SBP: systolic blood pressure; Total-C, total cholesterol; TG, triglycerides. LDL-C_cor and Total-C_cor represent the corrected LDL-C and Total-C. The ‘IncidenceRate’ shows the incidence rate of developing IS per 100,000 person-years in different subgroups. The ‘Heterogeneity’ shows the *P*-values of χ^2^ tests for HRs among subgroups. The ‘InteractionTerm’ shows the *P*-values of tests for the interaction terms between Lp(a) and the original risk factors.

**
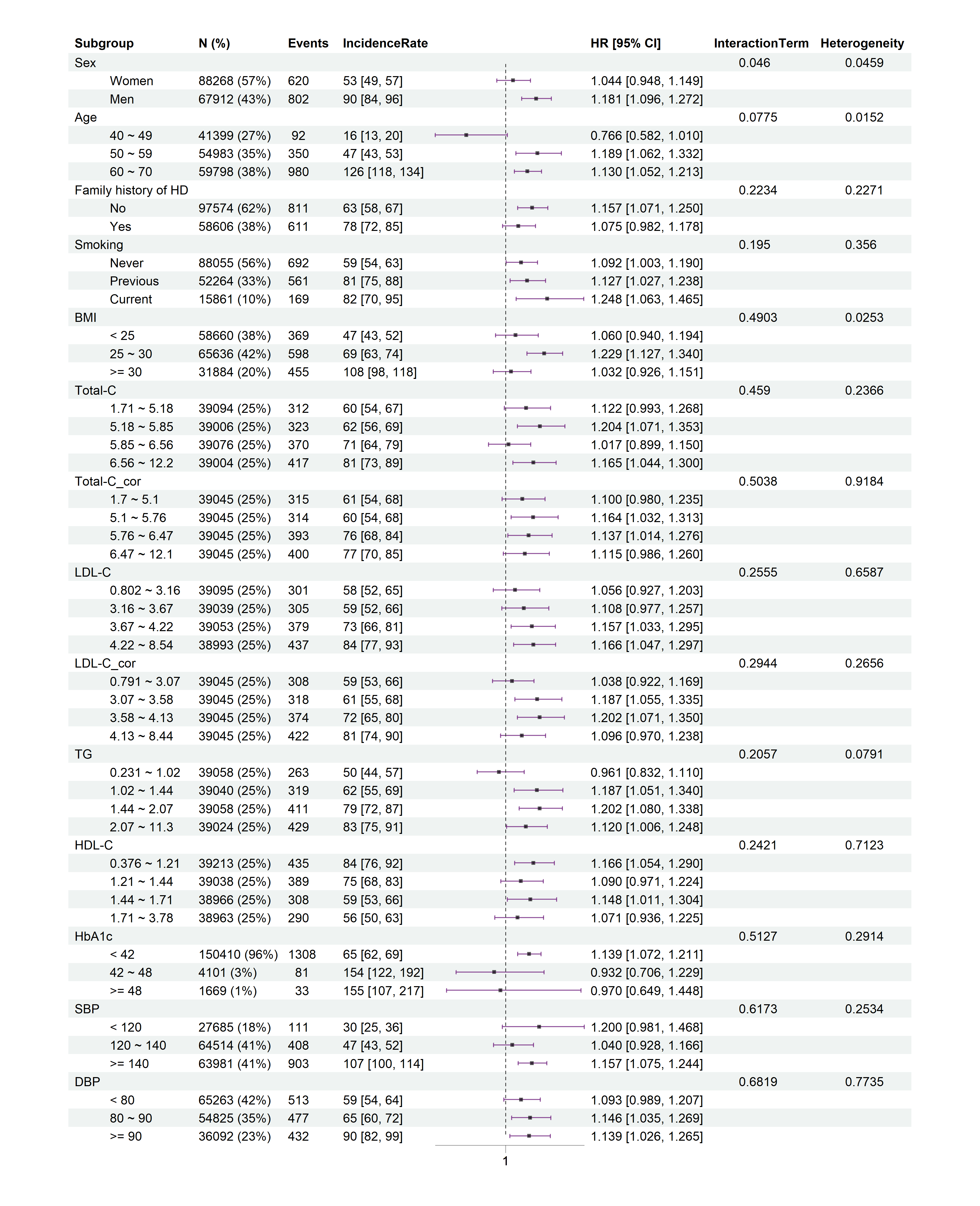
**

**Figure S6. The interactions between *LPA* GRS and the common cardiovascular risk factors, and the risk of developing calcific aortic valve stenosis (CAVS) for per one-SD increase of *LPA* GRS in subgroups**. Abbreviations: BMI, body mass index; CI, confidence interval; DBP, diastolic blood pressure; HbA1c: glycated haemoglobin; HD: heart disease; HDL-C, high-density lipoprotein cholesterol; HR, hazard ratio; LDL-C, low-density lipoprotein cholesterol; Lp(a), lipoprotein (a); SBP: systolic blood pressure; Total-C, total cholesterol; TG, triglycerides. LDL-C_cor and Total-C_cor represent the corrected LDL-C and Total-C. The ‘IncidenceRate’ shows the incidence rate of developing CAVS per 100,000 person-years in different subgroups. The ‘Heterogeneity’ shows the *P*-values of χ^2^ tests for HRs among subgroups. The ‘InteractionTerm’ shows the *P*-values of tests for the interaction terms between *LPA* GRS and the original risk factors.

**
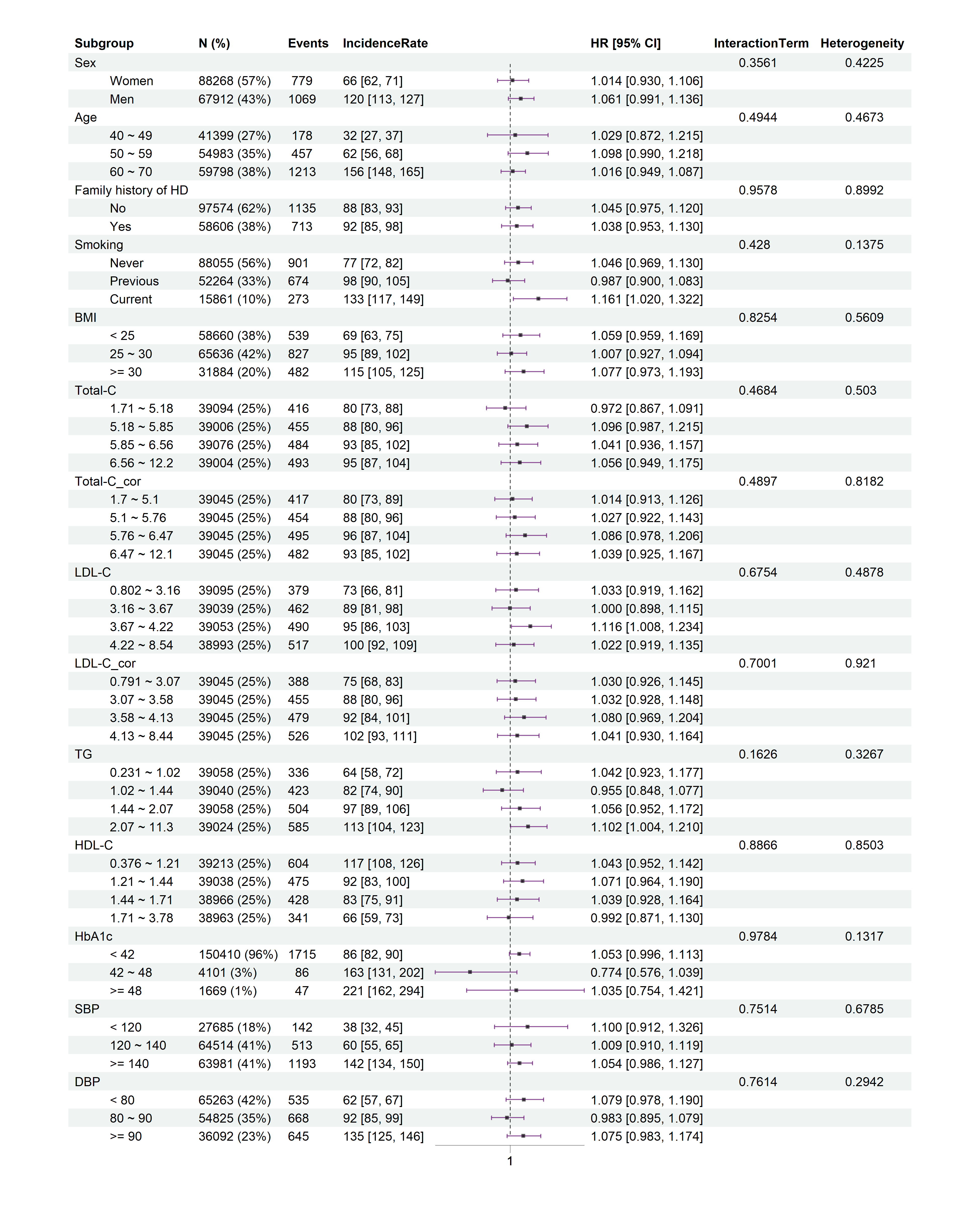
**

**Figure S7. The interactions between *LPA* GRS and the common cardiovascular risk factors, and the risk of developing ischemic stroke (IS) for per one-SD increase of *LPA* GRS in subgroups**. Abbreviations: BMI, body mass index; CI, confidence interval; DBP, diastolic blood pressure; HbA1c: glycated haemoglobin; HD: heart disease; HDL-C, high-density lipoprotein cholesterol; HR, hazard ratio; LDL-C, low-density lipoprotein cholesterol; Lp(a), lipoprotein (a); SBP: systolic blood pressure; Total-C, total cholesterol; TG, triglycerides. LDL-C_cor and Total-C_cor represent the corrected LDL-C and Total-C. The ‘IncidenceRate’ shows the incidence rate of developing IS per 100,000 person-years in different subgroups. The ‘Heterogeneity’ shows the *P*-values of χ^2^ tests for HRs among subgroups. The ‘InteractionTerm’ shows the *P*-values of tests for the interaction terms between *LPA* GRS and the original risk factors.

**
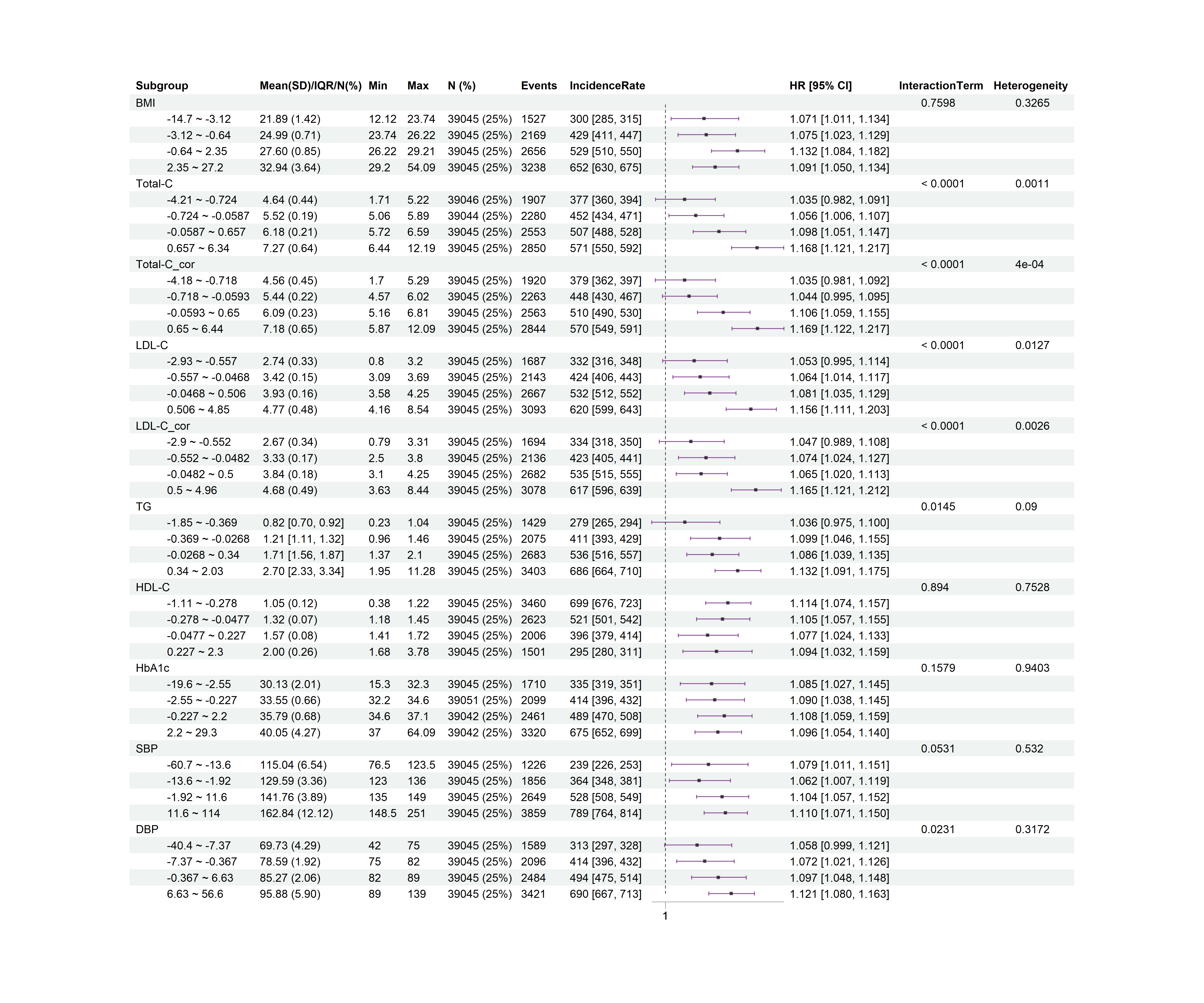
**

**Figure S8. The risk of developing coronary artery disease (CAD) for per one-SD increase of *LPA* GRS in subgroups defined by residual-risk factors.** Abbreviations: BMI, body mass index; CI, confidence interval; DBP, diastolic blood pressure; HbA1c: glycated haemoglobin; HD: heart disease; HDL-C, high-density lipoprotein cholesterol; HR, hazard ratio; LDL-C, low-density lipoprotein cholesterol; Lp(a), lipoprotein (a); SBP: systolic blood pressure; Total-C, total cholesterol; TG, triglycerides. LDL-C_cor and Total-C_cor represent the corrected LDL-C and Total-C. The ‘IncidenceRate’ shows the incidence rate of developing CAD per 100,000 person-years in different subgroups. The ‘Heterogeneity’ shows the *P*-values of χ^2^ tests for HRs among subgroups. The ‘InteractionTerm’ shows the *P*-values of tests for the interaction terms between *LPA* GRS and the residual risk factors.

**
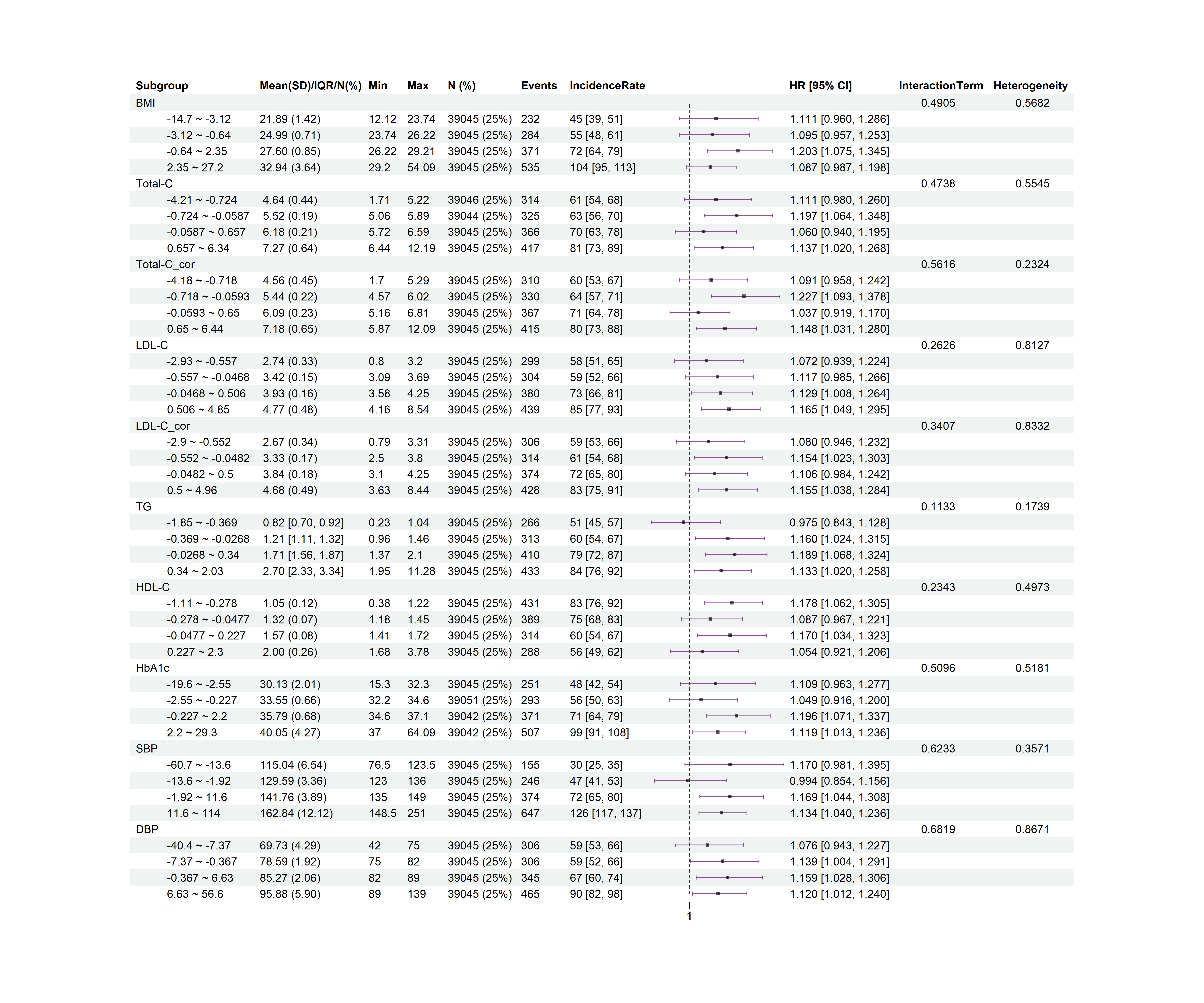
**

**Figure S9. The risk of developing calcific aortic valve stenosis (CAVS) for per one-SD increase of *LPA* GRS in subgroups defined by residual-risk factors.** Abbreviations: BMI, body mass index; CI, confidence interval; DBP, diastolic blood pressure; HbA1c: glycated haemoglobin; HD: heart disease; HDL-C, high-density lipoprotein cholesterol; HR, hazard ratio; LDL-C, low-density lipoprotein cholesterol; Lp(a), lipoprotein (a); SBP: systolic blood pressure; Total-C, total cholesterol; TG, triglycerides. LDL-C_cor and Total-C_cor represent the corrected LDL-C and Total-C. The ‘IncidenceRate’ shows the incidence rate of developing CAVS per 100,000 person-years in different subgroups. The ‘Heterogeneity’ shows the *P*-values of χ^2^ tests for HRs among subgroups. The ‘InteractionTerm’ shows the *P*-values of tests for the interaction terms between *LPA* GRS and the residual risk factors.

**
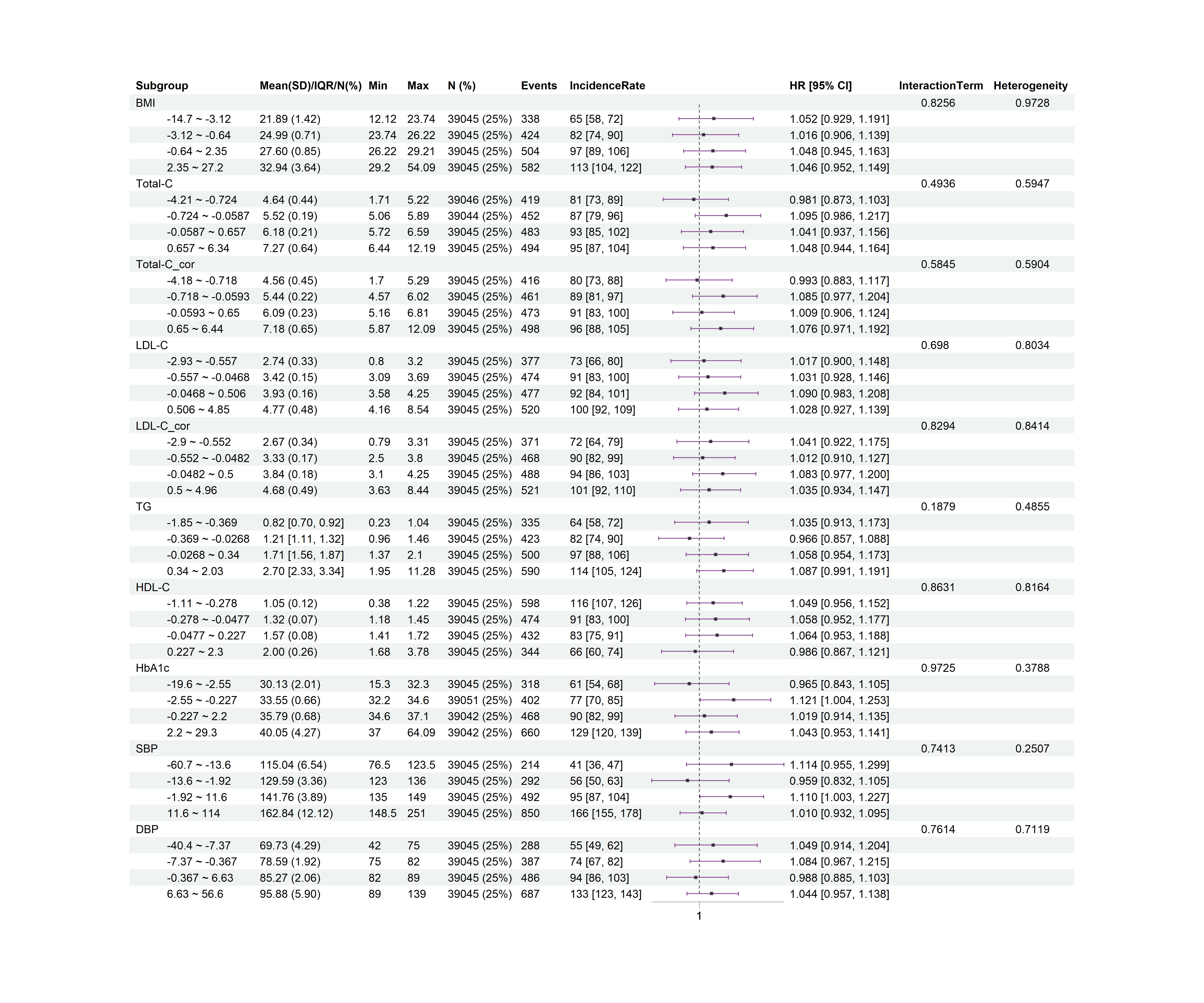
**

**Figure S10. The risk of developing ischemic stroke (IS) for per one-SD increase of *LPA* GRS in subgroups defined by residual-risk factors.** Abbreviations: BMI, body mass index; CI, confidence interval; DBP, diastolic blood pressure; HbA1c: glycated haemoglobin; HD: heart disease; HDL-C, high-density lipoprotein cholesterol; HR, hazard ratio; LDL-C, low-density lipoprotein cholesterol; Lp(a), lipoprotein (a); SBP: systolic blood pressure; Total-C, total cholesterol; TG, triglycerides. LDL-C_cor and Total-C_cor represent the corrected LDL-C and Total-C. The ‘IncidenceRate’ shows the incidence rate of developing IS per 100,000 person-years in different subgroups. The ‘Heterogeneity’ shows the *P*-values of χ^2^ tests for HRs among subgroups. The ‘InteractionTerm’ shows the *P*-values of tests for the interaction terms between *LPA* GRS and the residual risk factors.

**Table S1. Genetic variants used in the MR analysis and *LPA* genetic risk score obtained from Burgess et al. ^(1)^.**

| **Chromosome: PositionGRCh37/hg19** | **rsID** | **Effect Allele** | **Other Allele** | **MAF** | **Conditional Beta** | **Conditional SE** |
| --- | --- | --- | --- | --- | --- | --- |
| 6:160997118 | rs74617384 | T | A | 0.072 | 42.4 | 0.5 |
| 6:161013013 | rs140570886 | C | T | 0.011 | 80.2 | 0.8 |
| 6:161017363 | rs73596816 | A | G | 0.034 | 19.2 | 0.6 |
| 6:160891897 | rs182443492 | A | C | 0.009 | 36.8 | 1.0 |
| 6:161032800 | rs369686024 | A | G | 0.014 | 19.2 | 0.8 |
| 6:161089307 | rs56393506 | T | C | 0.169 | 12.4 | 0.4 |
| 6:160831796 | rs151135411 | A | G | 0.001 | 69.5 | 2.7 |
| 6:161292838 | rs145099029 | C | A | 0.003 | 17.8 | 1.8 |
| 6:160998199 | rs41267813 | A | G | 0.001 | -58.8 | 2.9 |
| 6:160890350 | rs6916433 | T | A | 0.14 | -4.7 | 0.3 |
| 6:161137990 | rs783147 | A | G | 0.45 | -2.0 | 0.3 |
| 6:160953137 | rs41266379 | C | T | 0.02 | 7.1 | 0.7 |
| 6:160954800 | rs143461353 | T | C | 0.008 | 13.1 | 1.0 |
| 6:160942926 | rs142126734 | A | G | 0.049 | 7.5 | 0.5 |
| 6:160899049 | rs139609547 | - | A | 0.054 | 4.4 | 0.4 |
| 6:161162290 | rs1835346 | G | A | 0.022 | 5.2 | 0.7 |
| 6:161159366 | rs4252152 | G | T | 0.014 | 9.1 | 0.9 |
| 6:161078894 | rs79246098 | C | T | 0.01 | 6.2 | 0.9 |
| 6:160966559 | rs139145675 | A | G | 0.001 | -22.5 | 2.4 |
| 6:161022107 | rs41259144 | T | C | 0.011 | -9.6 | 0.8 |
| 6:161012805 | rs9456551 | C | T | 0.35 | 3.6 | 0.2 |
| 6:160953642 | rs41267809 | G | A | 0.022 | -6.6 | 0.6 |
| 6:161257953 | rs34371670 | T | C | 0.016 | -8.4 | 0.7 |
| 6:161070653 | rs41269876 | A | C | 0.028 | -8.2 | 0.6 |
| 6:160909667 | rs141834709 | A | T | 0.009 | 8.7 | 1.0 |
| 6:161162406 | rs4252170 | C | T | 0.082 | 3.2 | 0.4 |
| 6:161251940 | rs138491411 | G | A | 0.012 | 5 | 0.8 |
| 6:160720804 | rs183815886 | C | G | 0.003 | 14.8 | 1.8 |
| 6:160847571 | rs117446263 | A | G | 0.022 | -5.2 | 0.6 |
| 6:160543317 | rs200684404 | T | C | 0.000 | 67.7 | 9.2 |
| 6:160493099 | rs200144324 | T | C | 0.000 | 81.5 | 11.3 |
| 6:161087652 | rs77337569 | G | T | 0.013 | 5.2 | 0.8 |
| 6:161214526 | rs186418835 | A | G | 0.004 | -9.7 | 1.5 |
| 6:161177443 | rs117534432 | T | C | 0.036 | 3.3 | 0.5 |
| 6:161011999 | rs200376184 | C | G | 0.001 | 17.5 | 2.7 |
| 6:161189071 | rs11753588 | A | G | 0.109 | -2.4 | 0.3 |
| 6:161285760 | rs4709474 | G | A | 0.49 | 1.7 | 0.2 |
| 6:161031132 | rs191690882 | A | G | 0.002 | -13.2 | 1.9 |
| 6:161255668 | rs182349273 | G | A | 0.000 | 34.4 | 5.7 |
| 6:161088956 | rs75274517 | A | G | 0.01 | -6.5 | 1.0 |
| 6:160825930 | rs143365644 | T | A | 0.035 | 3.7 | 0.5 |
| 6:161135746 | rs139389770 | G | T | 0.011 | -5.2 | 0.9 |
| 6:161250301 | rs140606700 | G | A | 0.007 | 6.4 | 1.2 |

**Table S2. The joint effects (hazard ratios [95% CI]) between Lp(a) and LDL-C, Total-C, and TG on coronary artery disease**

|  |  | **Lp(a) levels** | | |
| --- | --- | --- | --- | --- |
|  |  | **< 30 mg/dL** | **30 ~ 50 mg/dL** | **≥ 50 mg/dL** |
| **Total-C (mmol/L)** |  |  |  |  |
|  | 1.71 ~ 5.17 | Reference | 0.99 [0.84, 1.18] | 1.18 [1.02, 1.37] |
|  | 5.17 ~ 5.83 | 1.13 [1.04, 1.22] | 1.34 [1.15, 1.56] | 1.27 [1.11, 1.45] |
|  | 5.83 ~ 6.54 | 1.18 [1.09, 1.27] | 1.52 [1.32, 1.76] | 1.53 [1.37, 1.72] |
|  | 6.54 ~ 12.2 | 1.35 [1.25, 1.45] | 1.68 [1.46, 1.94] | 1.94 [1.75, 2.16] |
| **LDL-C (mmol/L)** |  |  |  |  |
|  | 0.802 ~ 3.15 | Reference | 1.04 [0.86, 1.25] | 1.22 [1.03, 1.43] |
|  | 3.15 ~ 3.66 | 1.15 [1.06, 1.25] | 1.23 [1.04, 1.45] | 1.25 [1.10, 1.44] |
|  | 3.66 ~ 4.21 | 1.28 [1.18, 1.38] | 1.59 [1.38, 1.84] | 1.59 [1.41, 1.79] |
|  | 4.21 ~ 8.50 | 1.47 [1.36, 1.59] | 1.93 [1.69, 2.20] | 2.13 [1.92, 2.36] |
| **TG**  **(mmol/L)** |  |  |  |  |
|  | 0.231 ~ 1.01 | Reference | 1.01 [0.83, 1.23] | 1.22 [1.05, 1.42] |
|  | 1.01 ~ 1.43 | 1.16 [1.07, 1.27] | 1.35 [1.14, 1.60] | 1.63 [1.44, 1.86] |
|  | 1.43 ~ 2.07 | 1.36 [1.25, 1.48] | 1.68 [1.46, 1.95] | 1.69 [1.49, 1.91] |
|  | 2.07 ~ 11.30 | 1.57 [1.45, 1.70] | 2.02 [1.77, 2.32] | 2.18 [1.95, 2.45] |

Abbreviations: CI, confidence interval; Lp(a), lipoprotein (a); LDL-C, low-density lipoprotein cholesterol; Total-C, total cholesterol; TG, triglycerides.

# **References**

1. Burgess S., Ference B. A., Staley J. R., Freitag D. F., Mason A. M., Nielsen S. F., et al. Association of LPA Variants With Risk of Coronary Disease and the Implications for Lipoprotein(a)-Lowering Therapies: A Mendelian Randomization Analysis. JAMA Cardiol. 2018;3(7):619-27.

2. Bycroft C., Freeman C., Petkova D., Band G., Elliott L. T., Sharp K., et al. The UK Biobank resource with deep phenotyping and genomic data. Nature. 2018;562(7726):203-9.

3. McCarthy S., Das S., Kretzschmar W., Delaneau O., Wood A. R., Teumer A., et al. A reference panel of 64,976 haplotypes for genotype imputation. Nat Genet. 2016;48(10):1279-83.

4. Consortium Uk K., Walter K., Min J. L., Huang J., Crooks L., Memari Y., et al. The UK10K project identifies rare variants in health and disease. Nature. 2015;526(7571):82-90.

5. Genomes Project Consortium, Auton A., Brooks L. D., Durbin R. M., Garrison E. P., Kang H. M., et al. A global reference for human genetic variation. Nature. 2015;526(7571):68-74.

6. Smith G. D., Ebrahim S. 'Mendelian randomization': can genetic epidemiology contribute to understanding environmental determinants of disease? Int J Epidemiol. 2003;32(1):1-22.

7. Davies N. M., Holmes M. V., Davey Smith G. Reading Mendelian randomisation studies: a guide, glossary, and checklist for clinicians. BMJ. 2018;362:k601.

8. Pierce B. L., Burgess S. Efficient design for Mendelian randomization studies: subsample and 2-sample instrumental variable estimators. Am J Epidemiol. 2013;178(7):1177-84.

9. Burgess S., Scott R. A., Timpson N. J., Davey Smith G., Thompson S. G., Consortium Epic- InterAct. Using published data in Mendelian randomization: a blueprint for efficient identification of causal risk factors. Eur J Epidemiol. 2015;30(7):543-52.

10. Burgess S., Butterworth A., Thompson S. G. Mendelian randomization analysis with multiple genetic variants using summarized data. Genet Epidemiol. 2013;37(7):658-65.

11. Nikpay M., Goel A., Won H. H., Hall L. M., Willenborg C., Kanoni S., et al. A comprehensive 1,000 Genomes-based genome-wide association meta-analysis of coronary artery disease. Nat Genet. 2015;47(10):1121-30.

12. Malik R., Chauhan G., Traylor M., Sargurupremraj M., Okada Y., Mishra A., et al. Multiancestry genome-wide association study of 520,000 subjects identifies 32 loci associated with stroke and stroke subtypes. Nat Genet. 2018;50(4):524-37.

13. Westerman Kenneth E., Pham Duy T., Hong Liang, Chen Ye, Sevilla-González Magdalena, Sung Yun Ju, et al. GEM: scalable and flexible gene–environment interaction analysis in millions of samples. Bioinformatics. 2021;37(20):3514-20.

14. Kurki Mitja I., Karjalainen Juha, Palta Priit, Sipilä Timo P., Kristiansson Kati, Donner Kati, et al. FinnGen: Unique genetic insights from combining isolated population and national health register data. medRxiv. 2022:2022.03.03.22271360.

15. Hoaglin D. C. We know less than we should about methods of meta-analysis. Res Synth Methods. 2015;6(3):287-9.

16. Bowden J., Davey Smith G., Haycock P. C., Burgess S. Consistent Estimation in Mendelian Randomization with Some Invalid Instruments Using a Weighted Median Estimator. Genet Epidemiol. 2016;40(4):304-14.

17. Burgess S., Thompson S. G. Interpreting findings from Mendelian randomization using the MR-Egger method. Eur J Epidemiol. 2017;32(5):377-89.

18. Bowden J., Davey Smith G., Burgess S. Mendelian randomization with invalid instruments: effect estimation and bias detection through Egger regression. Int J Epidemiol. 2015;44(2):512-25.
